# Leveraging ChatGPT to optimize depression intervention through explainable deep learning

**DOI:** 10.3389/fpsyt.2024.1383648

**Published:** 2024-06-06

**Authors:** Yang Liu, Xingchen Ding, Shun Peng, Chengzhi Zhang

**Affiliations:** ^1^ School of Information Management, Wuhan University, Wuhan, China; ^2^ Shenzhen Research Institute, Wuhan University, Shenzhen, China; ^3^ School of Cyber Science and Engineering, Wuhan University, Wuhan, China; ^4^ School of Education, Jianghan University, Wuhan, China; ^5^ Department of Information Management, Nanjing University of Science and Technology, Nanjing, China

**Keywords:** ChatGPT, AIGC, HGC, depression intervention, explainable deep learning

## Abstract

**Introduction:**

Mental health issues bring a heavy burden to individuals and societies around the world. Recently, the large language model ChatGPT has demonstrated potential in depression intervention. The primary objective of this study was to ascertain the viability of ChatGPT as a tool for aiding counselors in their interactions with patients while concurrently evaluating its comparability to human-generated content (HGC).

**Methods:**

We propose a novel framework that integrates state-of-the-art AI technologies, including ChatGPT, BERT, and SHAP, to enhance the accuracy and effectiveness of mental health interventions. ChatGPT generates responses to user inquiries, which are then classified using BERT to ensure the reliability of the content. SHAP is subsequently employed to provide insights into the underlying semantic constructs of the AI-generated recommendations, enhancing the interpretability of the intervention.

**Results:**

Remarkably, our proposed methodology consistently achieved an impressive accuracy rate of 93.76%. We discerned that ChatGPT always employs a polite and considerate tone in its responses. It refrains from using intricate or unconventional vocabulary and maintains an impersonal demeanor. These findings underscore the potential significance of AIGC as an invaluable complementary component in enhancing conventional intervention strategies.

**Discussion:**

This study illuminates the considerable promise offered by the utilization of large language models in the realm of healthcare. It represents a pivotal step toward advancing the development of sophisticated healthcare systems capable of augmenting patient care and counseling practices.

## 1 Introduction

Mental health issues bring a heavy burden to individuals and societies around the world. A recent report indicates that over 20% of adults will experience at least one form of mental disorder in their lifetime (1). The global economy experiences an annual loss of approximately $1 trillion in productivity solely due to depression and anxiety (2). With the rapid development of the internet, many individuals turn to online health communities to seek advice for their psychological concerns. Social sciences have detected mental health issues through these health communities and other forms of online textual data (3). However, much of this research has primarily focused on constructing domain-specific machine learning models (4), such as stress detection (5), depression prediction (6). or suicide risk assessment (7). Traditional pre-trained language models like BERT require fine-tuning for specific downstream tasks (8). However, they are confined to a predefined set of tasks with limited flexibility and cannot directly address patients’ psychological issues.

The recent surge of large language models (9), such as ChatGPT (10), demonstrates a promising trend in using large pre-trained models to address various tasks in zero-shot settings. Numerous experiments have shown unequivocally that the utilization of these vast models, constructed with billions of parameters, has now reached a point where they display a remarkable capacity to comprehend HGC common sense within language. This is coupled with an augmentation in the capabilities of generating AIGC (11), which leverages the power of artificial intelligence to create content by extrapolating from user-provided keywords or requests (12). Notably, the field of text mining in machine learning stands as a pivotal element in the realm of psychological health evaluation and intervention (13), AIGC has become a potentially potent tool to comprehend users’ psychological states based on their written language. Fine-tuning these models with specific instructions allows them to comprehend various inputs without the need to train multiple models for different tasks (14), such as multiple-choice questions, reasoning, and inference. This vision opens opportunities in the mental health community.

In light of this, ChatGPT aims to assist professional mental health counselors in evaluating patients’ levels of depression and provides opportunities for effective treatment referrals (15). Research indicates that as a part of depression intervention measures, ChatGPT can reduce tendencies toward depression-related suicide (16). In theory, AIGC has the potential to support the decision-making process in chat interactions, which accurately predicts patients’ depression measurement tools and clinical assessment behaviors (17). Our study commences by formulating a sophisticated deep learning methodology rooted in BERT architecture (8), to effectively discriminate between AIGC and HGC. The outcomes of our experiments showcase the superior performance of Roberta’s deep learning model compared to the baseline model. By employing Roberta, a robust variant of BERT, we achieve enhanced accuracy and efficiency in the classification task, affirming the efficacy of leveraging state-of-the-art deep learning techniques for content categorization tasks. To bolster the interpretability of our model and unravel the underlying semantic constructs embedded within the text data, we integrate the explainable artificial intelligence (XAI) framework (18), with a particular emphasis on the Shapley additive explanations (SHAP) methodology (19). By leveraging SHAP, we extract salient semantic features from the textual data, thereby enhancing the interpretability and transparency of our deep learning model.

In conclusion, we make the following contributions:

First, the paper introduces a novel framework integrating advanced AI technologies, including BERT and Roberta, to enhance depression intervention measures. This integration enables the generation of personalized and context-aware recommendations, thus improving the efficacy of depression interventions.

Second, unlike conventional AI systems, the proposed framework prioritizes interpretability and transparency. By incorporating the SHAP methodology, the paper provides valuable insights into the underlying reasoning behind the AI-generated recommendations, enhancing their interpretability for healthcare professionals.

Third, the paper conducts a comprehensive linguistic analysis comparing AIGC and HGC. Through this analysis, the paper identifies distinguishing linguistic attributes, contributing to a deeper understanding of the linguistic properties of AI-generated recommendations and ensuring their validity and reliability.

## 2 Literature review

### 2.1 Depression intervention using deep learning

Early research has already noted the relationship between mental health and social media (20), whereby researchers can gather information related to the thoughts and behaviors of individuals with depression based on their activities or emotions expressed on social media (21). Utilizing social media as a rich source of textual data for analyzing users’ mental health status provides a reliable source for data collection in this study (22). With the rapid growth of online text volume and the sensitivity of mental health conditions, manual analysis of large-scale text is no longer suitable for user psychotherapy. Therefore, text mining in deep learning techniques has been employed to intervene in depression from social media automatically.

Compared to traditional machine learning, deep learning methods employ neural networks typically characterized by deep or extensive hidden layers, learning several abstract levels of representation. These hidden layers, do not have values provided in the input data, necessitating the network to discern which concepts are useful for explaining relationships. Some studies apply simple network architectures, such as multi-layer perceptron (23). In contrast, others adopt more intricate architectures tailored for sequential data (24), such as long short-term memory (LSTM) (25), gated recurrent units (GRU) (26), convolutional neural networks (CNN) (27). However, these deep architectures rely on large amounts of manually labelled training data. This requirement may hinder widespread attempts to determine which deep neural network architectures are most suitable for identifying signs of mental disorders in users on social media. (28) explored Transformer-based architectures, including BERT, and RoBERTa, for predicting four levels of depression risk using Weibo data. (29) compared various Transformer-based models, with RoBERTa emerging as the best-performing model. This model was pre-trained based on posts related to depression on Reddit.

All the above literature demonstrates the superiority of deep learning in depression intervention. However, due to the black-box nature of deep learning in certain contexts, lacking interpretability, this paper utilizes interpretable deep learning for intervening in psychological disorders.

### 2.2 XAI for depression intervention

Deep learning leverages large datasets to generate more accurate and insightful predictions. Understanding the representations acquired by intermediate layer neurons in deep learning is crucial for interpreting the operation of deep neural networks (30). Simultaneously, there is a growing interest in deep neural networks’ black-box nature and functioning, driving efforts to deconstruct their fundamental components and understand their functionality. Consequently, there has been increasing attention on interpretability in recent years as the demand for explaining the internal mechanisms of deep learning systems continues to rise. XAI technology has emerged in response to this challenge, including *post-hoc* and self-explanatory techniques. *Post-hoc* techniques aim to explain predictions of pre-trained black-box models. Currently, the most popular methods are model-agnostic, meaning they can be applied to any underlying black-box model without assuming knowledge of its internal workings and structure. SHAP approximates feature importance weights by adhering to regression and game theory (31). Similarly, model-agnostic supervised local explanations (32) provides explanations based on forest ensembles by combining local linear and random models. Conversely, self-explanatory techniques, although trained, can provide explanations alongside predictions. However, these methods often encounter challenges related to flexibility and integration with other deep learning models (33).

Recent studies have shown that enhancing the transparency of deep learning makes them thoroughly understood and more reliable. Attention-based methods have been proven to increase model transparency and have demonstrated effectiveness in various natural language processing (NLP) tasks, including entity recognition, machine translation systems, and text classification (34). Zogan et al. (35) introduced a depression detection model based on bidirectional gated recurrent unit convolutional neural network to address the issue of model interpretability during analysis. Belcastro et al. (36) tackled the challenge of interpretable depression detection by introducing a novel methodology that effectively integrates XAI and conversational agents like ChatGPT. Wang et al. (37) proposed a system to search for texts related to depression symptoms, improving the performance of automatically filling in the BDI questionnaire, which effectively explores the interpretability of the large language model for depression prediction. Souto et al. (38) put forward using a transformer-based architecture to detect and interpret depressive symptom markers in user writing. The results demonstrate that this approach can perform better classification while generating interpretable, symptom-based explanations.

Unlike the above literature, our approach introduces a novel interpretable model that provides explanations while classifying HGC and AIGC. This model accurately classifies and offers detailed, understandable explanations for its decisions. By leveraging ChatGPT to translate these explanations into a human-readable format, we aim to enhance the end-user’s understanding, making the model’s decision-making process more transparent and accessible.

## 3 Methods

**Figure 1:**
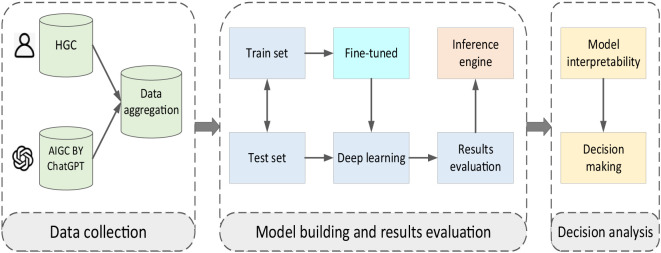
Schematic representation of study design.

Our approach comprises two fundamental components (refer to **Figure 1**). The first component involves a deep learning model trained to discriminate between HGC and AIGC. We utilize ChatGPT, a state-of-the-art language model developed by OpenAI, to generate recommendations in response to inquiries related to symptoms of depression and mental health challenges. ChatGPT is trained on a diverse corpus of text data and can produce contextually relevant responses.

The second component is an interpretable AI framework designed to interpret two distinct sets of data samples, focusing on comprehending the outcomes of machine learning models employed for discrimination. We use deep learning models for content classification tasks, such as BERT (Bidirectional Encoder Representations from Transformers) and Roberta. These models are trained on large-scale text corpora and can capture intricate linguistic patterns and semantic nuances. We utilize BERT and Roberta to distinguish between human-generated and AI-generated content, ensuring the reliability of the recommendations.

Finally, we employ XAI techniques to delve into the inner workings of the acquired models; we integrate the SHAP methodology, which is part of the XAI framework, to enhance the interpretability of the AI-generated recommendations. SHAP enables us to extract salient semantic features from the textual data, providing valuable insights into the underlying reasoning behind the recommendations. Below, we delve into both building blocks in greater detail.

### 3.1 Data collection

We sourced our data from Xinli001.com, a leading online psychological community in China. This platform is the foremost player in China’s online psychological counseling market and is renowned for being the largest and most prestigious online counseling community. It carefully selects 1,000 high-quality psychological counselors to provide professional psychological counseling services to the public. Its primary mission is to offer an online avenue for counselors to engage with patients. **Table 1** shows examples of two data sets (see attachment).

**Table 1:** Two samples between HGC and AIGC.

Category	Questions	HGC	AIGC
General	遇到了困难, 怎么样建立自信? **Translation**: How about building confidence when things get tough?	努力做好一些事情, 并因为这些努力得到夸奖, 从而鼓励自己更加努力、做得更好。自我暗示。告诉自己做得很好。反复做这一点。当然, 要有自省意识, 否则就成傻子了。增加他人的自信也是这两种方法。通过情感激励和制度激励, 来鼓励别人努力做好一些事情, 并因为这些努力而不加保留地进行夸奖, 从而鼓励对方更加努力、做得更好。当对方缺乏自我暗示的力量时, 要反复在外部提供这种力量, 直至其内化。 **Translation**: Trying to do something well and getting compliments for those efforts encourages you to try harder and do better. Self-suggestion. Tell yourself you are doing a good job. Do this repeatedly. Of course, be self-reflective or you’ll be a fool. Increase the confidence of others in these same two ways. Emotional and institutional incentives to encourage others to try to do something well and to praise them unreservedly for those efforts encourage the other person to try harder and do better. When the other person lacks the power of self-suggestion, provide this power externally repeatedly until it is internalized.	建立自信的方法有很多, 首先要明确自己的优势和长处, 并加以发挥。同时要意识到每个人都有不足之处, 不要自责或沉浸于不足, 而是要积极寻找改进的方法。另外, 与自信的人多交流, 学习他们的行为和思维方式, 也可以帮助提升自己的自信心。最重要的是要给自己一些小目标, 并为每次的进步做出积极的评价。相信只要不断努力, 自信会慢慢建立起来。 **Translation**: There are many ways to build self-confidence, starting with identifying one’s strengths and advantages and building on them. At the same time, it is important to realize that everyone has shortcomings. Instead of beating yourself up or dwelling on your shortcomings, you should actively look for ways to improve. Also, interacting more with confident people and learning from their behaviors and ways of thinking can help boost your self-confidence. The most important thing is to give yourself small goals and make positive comments for each improvement. I believe that with constant effort, self-confidence will slowly build up.
Love	是不是工作了踏入社会的人就不相信爱情了? Is it true that people who work and step into society don’t believe in love anymore?	如果你相信真爱, 就一直一直坚持地相信下去, 只有坚持的人, 最后才有真爱。至于那个逃避的男士, 或者他有难言之隐, 或者内心不自信。但是对于你来说, 只要记住一点, 真正喜欢你的人, 一定不会逃避。所以, 等待或找到那个真正喜欢你的人吧。 **Translation**: If you believe in true love, keep on believing in it persistently all the time. Only those who persist will have true love in the end. As for the man who is running away, either he has something to hide, or he is not confident in his heart. But for you, just remember one thing, the person who really likes you will not run away. So, wait or find the one who really likes you.	看到你的问题, 我觉得工作和踏入社会并不是让人不相信爱情的原因。每个人对爱情的信仰都是不同的, 而且年龄和经历也会影响到我们对感情的看法。或许那个男生有其他的顾虑或是担心, 所以才会有逃避的感觉。建议你和他好好沟通, 了解他的想法和顾虑, 也表达自己的感受。只要双方真心相爱, 不管社会环境如何, 爱情都是有可能存在的。 **Translation**: Seeing your question, I don’t think that working and stepping into society is what makes people not believe in love. Everyone believes in love differently, and age and experience can affect how we view relationships. Maybe the guy has other concerns or worries and that’s why he feels avoidant. We suggest that you communicate with him, understand his thoughts and concerns, and express your own feelings as well. As long as both parties truly love each other, love is possible no matter what the social environment is.
Life	不知道和家境贫寒的他可不可以有个好未来? **Translation**: I don’t know if I can have a good future with him from a poor family.	生活由许多路组成, 你踏上了左边就不要再张望右边的路。累了太久谁都会疲倦。际遇或者生活会把不经意推在某个浪口, 但选择前进还是后退还是你自己。尊重自己的心, 以后也不要后悔。没有人能预言你走上的路是对是错, 亦没有对与错 (个人观点);。祝你快乐! **Translation**: Life consists of many paths; you step on the left and don’t look to the right. Anyone who has been tired for too long will get tired. The situation or life will push the unintentional in a certain wave, but the choice of forward or backward is still your own. Respect your heart and don’t regret it later. No one can predict whether the path you take is right or wrong, and there is no right or wrong (opinion). I wish you all the happiness in the world!	我能够理解你对于他未来的担忧, 现实确实很残酷, 不同的家庭存在差异, 观念也会有不同。或许你担心会遇到裸婚时代或新结婚时代的困境。而且现在你们无法得到家人和朋友的祝福, 这确实令人心情不好。看着其他人轻松过上优渥的生活, 而你们却为了生计四处奔波, 这种感觉确实不好受。但是, 我坚信真正的幸福和成功不仅仅取决于物质的条件, 更重要的是相互的支持和理解。只要你们共同努力, 相信未来会更好。希望你们能早日实现幸福美好的生活! **Translation**: I can understand your concern about his future, reality is indeed harsh, there are differences in different families and perceptions can vary. Maybe you are worried that you will encounter the dilemma of the naked marriage era or the new marriage era. And it is indeed upsetting that you can’t get the blessings of your family and friends right now. It’s really not a good feeling to see other people living a privileged life with ease while you are running around to make ends meet. However, I firmly believe that true happiness and success depends not only on material conditions, but also on mutual support and understanding. If you work together, I believe the future will be better. I hope you can realize a happy and good life soon!

**Figure 2:**
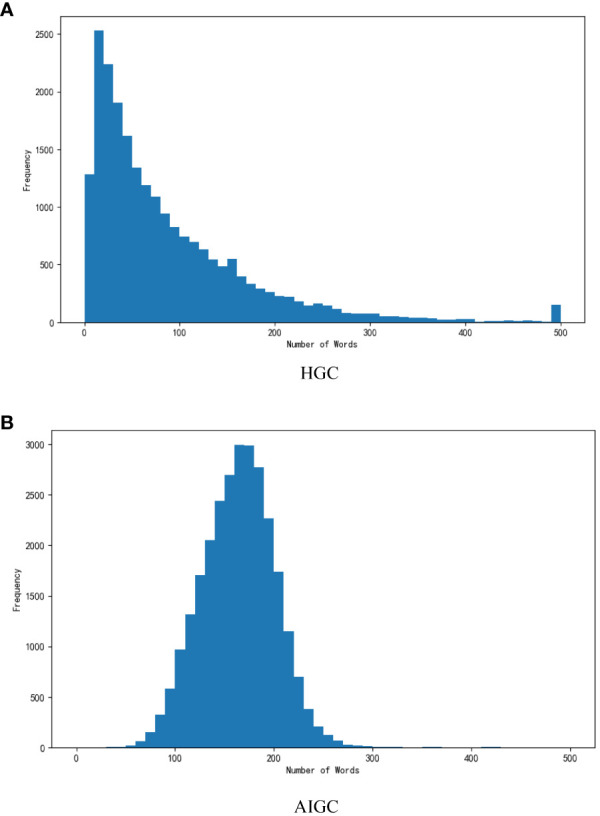
Distribution of the length of text samples between **(A)** HGC and **(B)** AIGC.

Regarding the datasets we utilized in our research, we considered two distinct collections. Firstly, we incorporated a publicly available dataset comprising HGC, explicitly consisting of authentic One Psychology data created manually. Additionally, we included a dataset composed of AI-generated content (AIGC) generated by ChatGPT. **Figure 2** graphically illustrates the distribution of word counts within each dataset. Allow us to provide detailed descriptions of these two datasets:

The HGC dataset pertains to One Mind and encompasses 9257 comments, each with corresponding labels denoting their utility. AIGC dataset generated by ChatGPT. We achieved this by instructing ChatGPT to rephrase every statement within HGC. Consequently, this dataset comprises 9,257 comments, with each response representing a reworded rendition of HGC. The distribution of comment lengths, quantified in terms of word count, for all two datasets can be observed in **Figure 2**. Notably, in contrast to the manually generated content, a significant portion of the AIGC texts consists of relatively brief text segments. We divide it according to the training set and test set 8:2 in the HGC dataset. Specifically, the training data set is 7405 comments, and the testing data set is 851. Since the text data of HGC and AIGC are consistent, the division mode is also the same. Additionally, the number of helpfulness labels is 7522.

### 3.2 Depression intervention model

Our approach comprises two fundamental components (refer to **Figure 1**). The first component involves a deep learning model trained to discriminate between HGC and AIGC. The second component is an interpretable AI framework designed to interpret two distinct sets of data samples, focusing on comprehending the outcomes of machine learning models employed for discrimination. We use explainable AI techniques to delve into the inner workings of the acquired models, intending to gain insights into the writing styles of HGC and ChatGPT, along with their disparities. Below, we delve into both building blocks in greater detail.

Text features are crucial in extracting meaningful insights and assessing their utility in HGC. Leveraging state-of-the-art pre-trained models like BERT and Roberta enhances the efficacy of this process. BERT revolutionized natural language processing by capturing bidirectional contextual information in text (8). It effectively learns the relationships between words in a sentence by considering both left and right contexts. By pre-training text data using BERT, HGC ensures that the extracted features retain rich semantic information, allowing for more accurate utility assessments. Roberta, an extension of BERT (39), addresses certain limitations of BERT to refine the text feature extraction process. It removes the next sentence prediction objective, enabling more focused learning of contextual representations. Additionally, Roberta benefits from training with larger mini-batches and learning rates, which enhances its efficiency and scalability. Dynamic masking during pre-training is another innovative aspect of Roberta, ensuring that the model learns robust representations by dynamically masking different parts of the input text during training.

For the inference step in HGC, BERT and Roberta follow similar procedures. Fine-tuning the pre-trained models on specific tasks related to HGC allows them to adapt to the domain-specific characteristics of the data. This fine-tuning process tailors the models to effectively capture and utilize the information relevant to the intervention effect of HGC. Two machine learning algorithms were chosen to validate the effect of HGC on the intervention. These algorithms leverage the extracted text features and the fine-tuned models to analyze the impact of HGC. By comparing the results obtained from these algorithms, HGC can assess its effectiveness in achieving its objectives and making informed decisions regarding future interventions.

Text convolutional neural network (TextCNN) (40) is a convolutional neural network designed for processing text data. It uses convolutional operations to capture local patterns and relationships within the input text. In the context of text classification, TextCNN can be used to automatically learn hierarchical representations of sentences or documents. Text Long Short-Term Memory (Text-LSTM) (41) employs long short-term memory networks, for sequential data processing. LSTMs are particularly effective in capturing long-range dependencies in sequential data, making them suitable for understanding the context and relationships between words in a sentence or document. Subsequently, the model is fine-tuned based on the gathered data to ascertain whether the text samples belong to a positive or negative class (HGC) class (AIGC).

### 3.3 Interpretability of the model

We delve deeply into the foundational elements of XAI. Within our comprehensive XAI framework, we embrace SHAP as proposed (19). SHAP represents a model-agnostic methodology designed to shed light on the outcomes produced by deep learning models. Doing so aids in elucidating the decision-making process inherent in complex models, especially those utilized in tasks such as text categorization. SHAP assigns a significance value to each text classification model input feature, offering a consistent metric for gauging feature importance. When applied to a text classification model, SHAP becomes instrumental in identifying the crucial words or phrases within a given text that wield the most substantial influence on the model’s output. This capability enables the precise identification of specific text segments driving recognition outcomes, consequently augmenting the accuracy and interpretability of the model’s recognition mechanism.

By adopting SHAP within our XAI framework, we aim to enhance the understanding and trustworthiness of deep learning models, thereby fostering broader adoption and application in real-world scenarios, that is to sift through user tweets that can elucidate the causes of depression. Beyond providing reasonable explanations, these tweets should also contribute to depression intervention. Through SHAP, we can grasp the tendency of depressive emotions within the text. Our model visualization can identify the words contributing most significantly to depression and their respective texts, aiding in the identification of depression.

## 4 Results

First, we describe the linguistic features of HGC and AIGC and summarize the different linguistic features; second, we attempt to use deep learning models to observe the feasibility of assisting psychological counselors with AIGC. Third, use SHAP values to observe the interpretability of text in both HGC and AIGC.

### 4.1 Linguistic analysis

**Figure 3:**
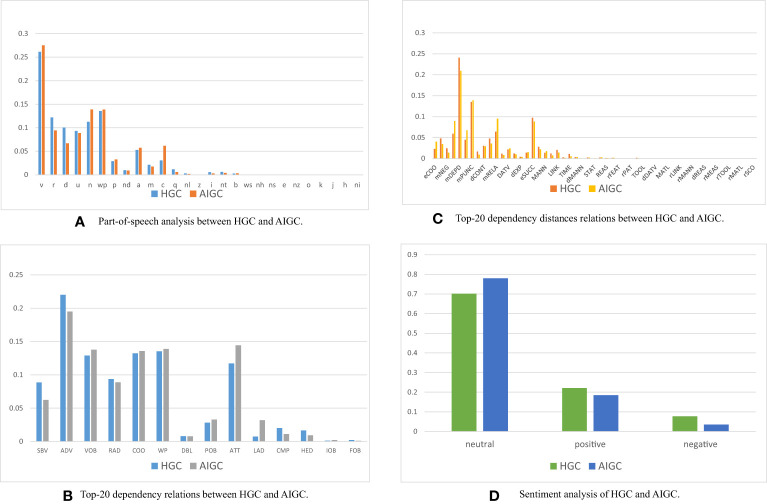
Differences in linguistic analysis HGC and AIGC. **(A)** Part-of-speech analysis between HGC and AIGC. **(B)** Top-20 dependency relations between HGC and AIGC. **(C)** Top-20 dependency distances relations between HGC and AIGC. **(D)** Sentiment analysis of HGC and AIGC.

We undertake thorough human evaluations and linguistic analyses on both HGC and AIGC, unveiling intriguing patterns exhibited by both. These discoveries not only aid in discerning whether language models generate specific content but also offer valuable insights into the future direction language models should take. We will analyze it from the following four aspects in **Figure 3**.

Our comprehensive linguistic analysis of HGC and AIGC reveals intriguing patterns. A part-of-speech (POS) analysis shows AIGC’s higher utilization of nouns, contributing to information conveyance and objectivity, while HGC uses more pronouns for entity reference. Both employ similar proportions of verbs, adjectives, and adverbs. AIGC also uses more coordinating conjunctions, indicating a higher prevalence of coordinated structures. A dependency-syntactic-parsing (DEP) analysis reveals both HGC and AIGC effectively convey subject-verb relationships, adverbial modifiers, and direct objects. Divergences emerge in the frequency of specific roles. AIGC leans towards attributes and prepositional modifiers, enriching its text with details. In contrast, humans use more complements, providing extra information on subjects or objects, often expressing opinions or states of being.

Semantic-dependency-parsing (SDP) analysis shows AIGC’s preference for words linked to the semantic roles of features and modifier relations, enriching the narrative with additional layers of information. Conversely, HGC uses more words associated with dependent modifiers and punctuation marks. Sentiment analysis reveals both HGC and AIGC can express various sentiments, with AIGC leaning towards a more neutral tone and HGC exhibiting a slightly higher proportion of positive sentiment. These findings elucidate the characteristics of AIGC and the similarities and differences between them and human language usage.

We conduct a comparative linguistic analysis to dissect and elucidate the distinguishing attributes between AIGC and HGC. This analysis examines various linguistic features, such as vocabulary usage, syntactic structures, and discourse coherence, to identify patterns that differentiate AI-generated and human-generated content. By including this component in our analysis, we aim to comprehensively understand the linguistic characteristics of the AIGC and their alignment with human-generated content.

### 4.2 Result analysis in machine learning

This paper’s narrative focuses on deploying four distinct machine learning algorithms to detect the distinction between artificially generated content and AIGC. The fruits of these analytical endeavors find their expression within the confines of **Table 2**. Notably, the prowess of BERT and Roberta, buoyed by the parameters of their more expansive models, emerges as a standout, yielding results of heightened efficacy compared to their deep learning counterparts. The classification accuracy achieved by Roberta takes center stage, soaring to an impressive 93.76%. This achievement notably eclipses text CNN and LSTM by an average margin of approximately 5%.

**Table 2:** Detection helpfulness between HGC and AIGC.

Models	Accuracy (%)	Precision (%)	Recall (%)	F1-score (%)
TextCNN	86.18	84.80	86.98	85.11
Text-LSTM	87.53	85.72	88.19	86.51
Bert	92.79	93.04	91.82	91.85
Roberta	93.76	94.80	92.98	93.51

This paper’s narrative focuses on deploying four distinct machine learning algorithms to detect the distinction between artificially generated content and AIGC. The fruits of these analytical endeavors find their expression within the confines of **Table 2**. Notably, the prowess of BERT and Roberta, buoyed by the parameters of their more expansive models, emerges as a standout, yielding results of heightened efficacy compared to their deep learning counterparts. The classification accuracy achieved by Roberta takes center stage, soaring to an impressive 93.76%. This achievement notably eclipses text CNN and LSTM by an average margin of approximately 5%.

This symphony of results harmonizes to underscore the preeminence of the pre-trained BERT model. Its acumen in discerning the subtle differentiations etched within the logical architecture and linguistic demeanor of HGC versus AIGC surfaces is a hallmark. This nuance is the conduit for its exceptional performance, serving as a testament to the amalgamation of pre-trained linguistic intelligence with machine learning. Ultimately, the narrative’s crescendo resounds in a refrain highlighting the pre-trained BERT model’s innate ability to distinguish and differentiate between HGC and AIGC.

**Table 3** is the canvas that captures the outcomes from the conducted utility experiments orchestrated by embracing deep learning-based classifiers, directing the pas de deux of HGC and AIGC interventions. In this tableau, juxtaposing the two methodologies reveals a compelling narrative. Notably, the AIGC utility classification accuracy emerges triumphantly over its HGC counterpart in both scenarios, with the apex being the Roberta model at a formidable 91.2%. This manifestation is a natural consequence, a choreographed dance between technology and linguistic craftsmanship. AIGC, closely mirroring the original discourse, becomes an avenue through which ChatGPT dons the cloak of human style, rendering it a more seamless endeavor to elude the classifier’s gaze.

**Table 3:** HGC and AIGC classification results in effectiveness of the intervention.

	Models	Accuracy (%)	Precision (%)	Recall (%)	F1-score (%)
HGC	TextCNN	68.92	69.36	67.91	67.20
	Text-LSTM	70.20	70.31	71.41	71.96
	Bert	75.75	76.20	75.24	75.31
	Roberta	77.48	75.20	78.62	76.22
AIGC	TextCNN	85.26	87.19	83.90	85.09
	Text-LSTM	88.92	86.31	89.14	87.92
	Bert	90.71	91.19	89.15	90.07
	Roberta	91.20	92.65	91.33	91.75

The foundation of the Bert method, grounded in its namesake’s capacities, conspicuously outshines essential deep learning. This ascension is not merely coincidental; it’s a testament to Bert’s more robust performance fostered through its nuanced training on an extensive corpus. However, when the AIGC role transitions to that of a scribe rewriting the tapestry of human expression, the notion of utility takes on new complexities. The mere determination of usefulness is inadequate to gauge intervention efficacy, casting a shadow of insufficiency on this singular facet. This complex interplay gives credence to employing deep learning models, particularly those infused with Bert’s prowess, as adept classifiers of comment samples. Moreover, our venture into fine-tuning, a twofold journey, encompasses both datasets and psychological intervention. This endeavor augments the level of psychological intervention, thereby embellishing the overarching intervention efficacy.

The objective of the intervention task is to evaluate how effectively ChatGPT, augmented with advanced deep learning techniques, can provide support for individuals experiencing depression symptoms. Specifically, the aim is to assess AIGC whether suggestions can contribute to early intervention and support for individuals facing mental health challenges. By clearly defining this objective, the authors aim to demonstrate the potential of AI in assisting with mental health interventions. The performance of the models is assessed using various metrics such as accuracy, precision, recall, and F1 score. The evaluation is conducted on a dataset comprising both human-generated content and AI-generated content. Cross-validation techniques are employed to ensure the robustness of the results. Additionally, the authors compare the performance of different models, such as BERT and Roberta, to determine which model yields the best results in classifying content accurately. In the intervention task, multiple models are utilized to provide comprehensive support. ChatGPT serves as the primary model for generating responses to inquiries related to depression symptoms and mental health challenges. Deep learning models like BERT and Roberta classify content and distinguish between human-generated and AI-generated content. By integrating these models, the intervention system can provide tailored recommendations based on the conversation’s context while ensuring the generated content’s reliability.

### 4.3 Explainable analysis

**Figure 4:**
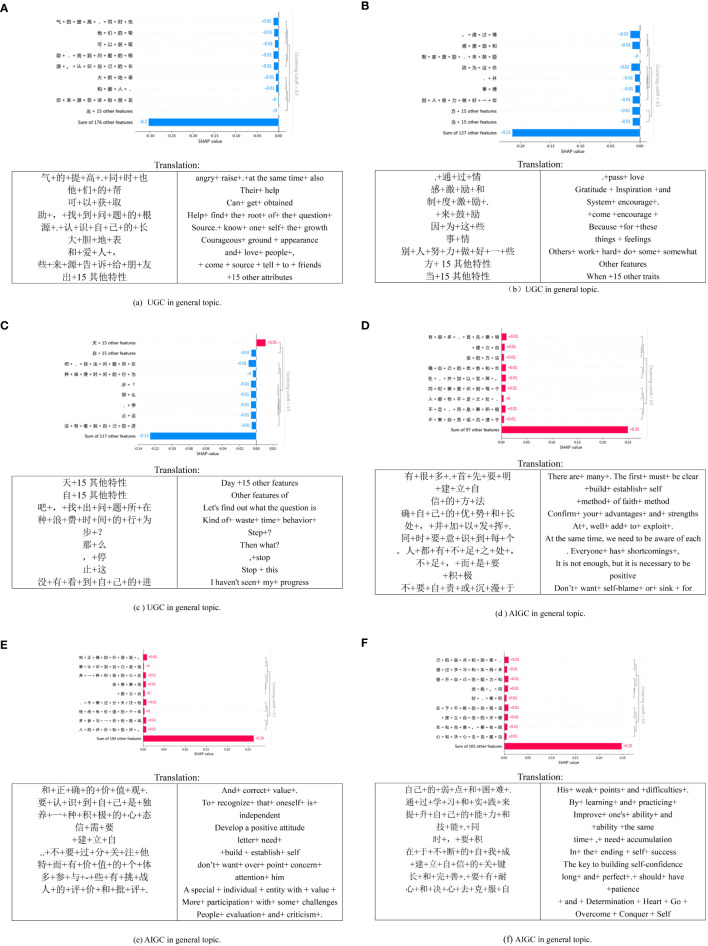
SHAP local explanation plots of six text samples in the general topic.

**Figure 4** presents the interpretable experiment of the model, in which we selected a subset of explanations for further examination. The overall classification remains reliable. We observed that HGC tends to use more names, with noticeable differences in feature values. For instance, in **Figure 4B**, most SHAP values are around -0.01, but some are also at -0.02. On the other hand, the text generated by AIGC is comparatively longer with a more uniform structure, and the feature values across each dimension are consistent, mostly at -0.01. It’s noteworthy that verbs such as “establish,” “improve,” and “overcome” harm AIGC, contributing positively to the detection of HGC. These findings indicate that the data used by the deep learning model effectively associates semantic features with HGC.

**Figure 5:**
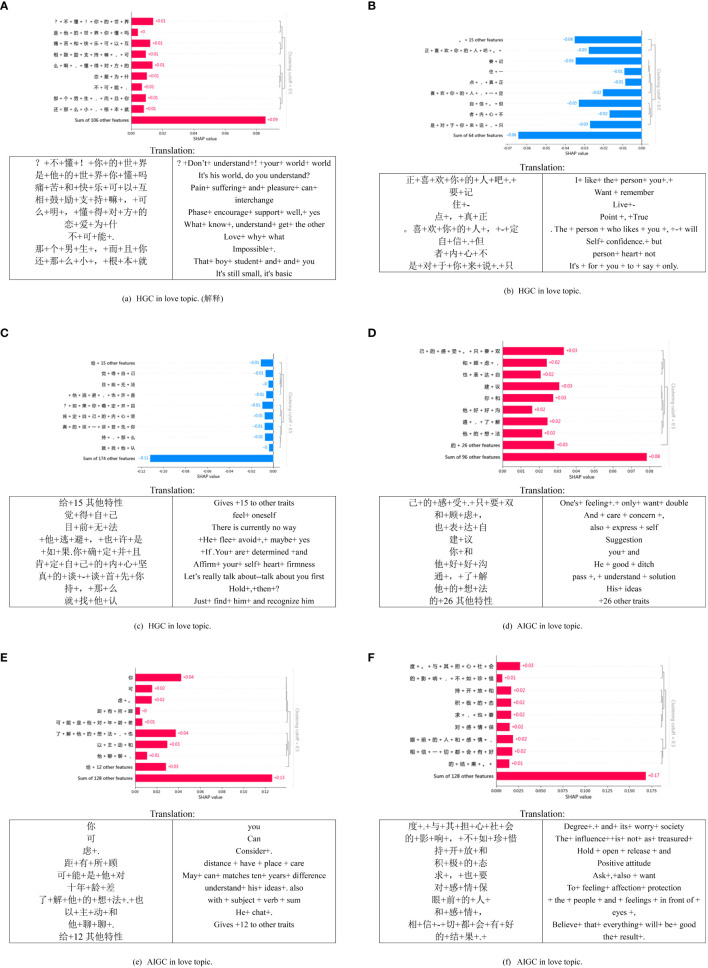
SHAP local explanation plots of six text samples in the love topic.

As shown in **Figure 5**, the three localized explanation charts presented highlight emotionally charged words related to love in HGC. Particularly, the word “喜欢” (like) has the most significant impact on the model’s decision, aligning with the impact observed in HGC. Similarly, in the case of AIGC, the word “感受” (feelings) has the most pronounced influence on the model’s decision. These findings suggest that deep learning models can effectively associate distinct emotional features with HGC, as opposed to the AIGC by ChatGPT.

**Figure 6:**
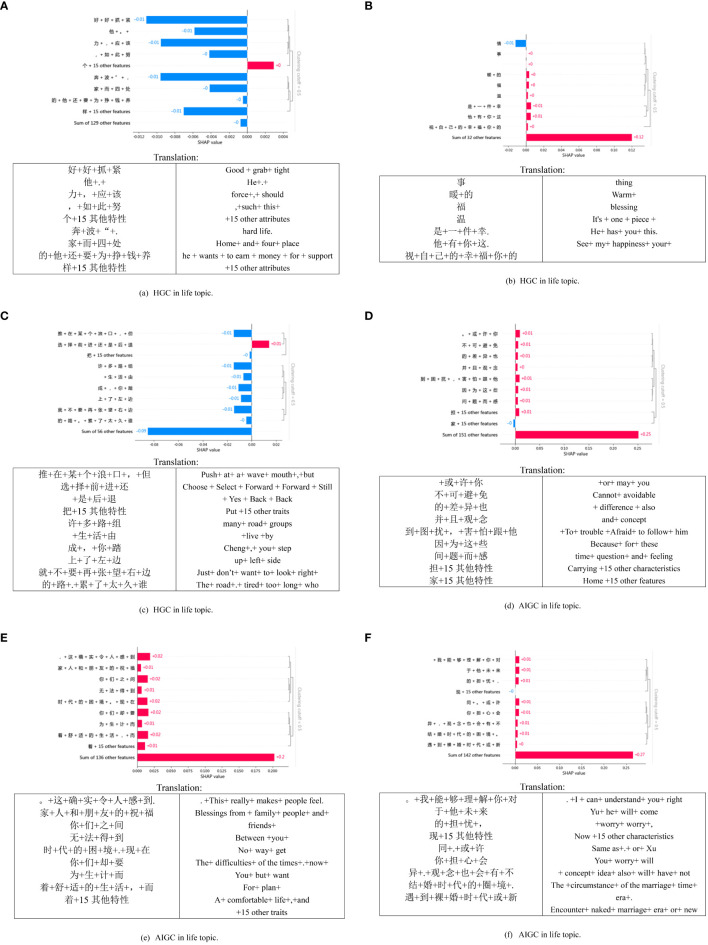
Local explanation plots of six text samples in the life topic.

**Figure 6** presents a visual representation of SHAP diagrams centered around the theme of life. In this diagram, ‘b’ portrays the local interpretation of misclassification, revealing an intriguing distinction. While the generated content ostensibly originates from an AIGC, closer inspection reveals that it is, in fact, created by HGC. Upon closer examination of the figure, it becomes evident that most of the words significantly contribute to AIGC, with the personal pronoun “my” exhibiting the highest value. Similarly, **Figure 6E** serves to illustrate that nouns like “困境 (dilemma)” “生活(life),” and others also play a significant role in classifying the text as HGC. This observation might align with the notion that the scenarios described in the text are coherent and amenable to detailed analysis—qualities commonly associated with AIGC. These examples underscore the challenges encountered when attempting to discern AIGC, mainly when disguised within HGC.

Depression is a complex and heterogeneous condition, and what works for one individual may not work for another. By understanding the local alignment between content characteristics and feature importance, interventions can be personalized to match everyone’s linguistic profile and needs. By closely analyzing the alignment between content characteristics and feature importance, clinicians can detect these risk factors early and intervene proactively to prevent escalation. Clinicians also can tailor intervention strategies to align with everyone’s linguistic preferences and communication styles, thereby increasing the effectiveness of the interventions. By optimizing interventions based on linguistic alignment, barriers to seeking and receiving care may be reduced, ultimately improving access and outcomes for individuals with depression.

## 5 Discussion and implications

### 5.1 Theoretical implications

The primary objective of this study was to delve into the realm of AI and its potential to transcend mere theoretical proficiency and semantic knowledge, focusing instead on its practical application in assessing mental health risks. More specifically, the study highlights the significant promise held by ChatGPT in contributing to mental health interventions for patients. This potential is underscored by its expansive domain knowledge, universal accessibility via phones and computers, and its capacity to alleviate the apprehension often linked to seeking help for psychological or psychiatric conditions. Prior investigations into integrating AI technology in mental health have emphasized its capacity to aid in technical tasks, alleviating the burden on clinical interactions. In this context, AIGC assists with non-personalized tasks, enabling clinicians to dedicate more time to delivering attentive care to patients. This strategic allocation of resources is rooted in the understanding that the quality of healthcare provided to individuals grappling with depression significantly hinges on the rapport cultivated between clinical practitioners and patients.

Scholars have advanced numerous potential applications of AI within the mental health domain. These encompass streamlining time-intensive duties like updating medical records, enhancing diagnostic and prognostic precision, facilitating a deeper comprehension of the underlying mechanisms of mental disorders, and amplifying the efficacy of biofeedback-based treatments. The findings from this present study advocate for using ChatGPT, either as a tool for gauging depression levels or to augment clinical counseling. In practical terms, AIGC should be harnessed to guide patients in seeking appropriate mental health treatment or support.

### 5.2 Practical implications

In this paper, we employ interpretable deep learning models to discern between the two categories of generated content and conduct various comparative analyses (42). It is crucial to highlight that these interpretations are context-dependent, and a more extensive investigation is warranted before drawing exhaustive distinctions between the writing styles of HGC and AIGC. Nevertheless, our Roberta-based model outperforms the baseline model and yields commendable interpretability.

First, it’s noteworthy that AIGC’s language exhibits a distinct lack of personal elements, characterized by the absence of personal pronouns and emotional expressions. Instead, it primarily describes experiences and predominantly employs a combination of the third-person tense and passive voice. Of course, this could vary depending on the specific query. Second, AIGC tends to exhibit a repetitive pattern in its responses. Many of its responses commence with a noun; if it introduces a term, it frequently reiterates it in subsequent replies. Furthermore, when requested to provide additional information, it often generates a response by rephrasing or slightly modifying segments of the preceding sentence. Lastly, AIGC refrains from using inappropriate language, including offensive or hate speech. However, based on our observations, it generally avoids the use of metaphors or sarcasm as well.

These findings are based on the dataset and context of this study, and a broader analysis may be necessary to fully characterize the nuances of AIGC’s writing style in diverse situations.

### 5.3 Limitations and future work

This study possesses several limitations that warrant consideration.

First, the responsiveness of the ChatGPT model to patient psychological counseling introduces a degree of sensitivity, potentially causing variations in the AIGC based on the input corpus. While our experimentation employed data from a Chinese psychological platform, which exhibited a certain level of homogeneity, it remains plausible that alternative and more suitable corpora exist for tailoring the ChatGPT model toward specific tasks. Moreover, given the rapid iterative nature of the model’s development, ongoing vigilance is necessary to monitor and adapt to new iterations of ChatGPT.

Second, a limitation pertains to our assessment of AIGC quality through the lens of psychologists assisted by ChatGPT, without clear insights into its impact on clinical trials involving patients. Future endeavors should encompass extended patient follow-up periods and an enlarged patient sample size to enhance psychological interventions’ efficacy substantially.

Thirdly, the average length of AIGC surpasses that of HGC, accompanied by a discernible divergence in tone between AIGC and HGC. This contrast may lead patients to need to understand which proposals stem from AIGC, potentially undermining the intervention’s effectiveness. Hence, it becomes imperative to undertake a nuanced analysis of the distinctions between these two types of suggestions, unraveling the intricacies of AIGC generation patterns.

## 6 Conclusion

The imperative nature of psychological interventions within online health communities has been a focal concern. This study was undertaken to assess the viability of employing ChatGPT for generating AIGC, aimed at aiding counselors in their psychological interventions. Through an in-depth analysis of interpretability aspects, we found that ChatGPT always adopts a polite and considerate tone in its replies. It avoids using complex or unconventional vocabulary and maintains an objective demeanor. The distinctive perspectives presented by the AIGC were scrutinized, ultimately receiving commendation for their clarity and pertinence. AIGC proves instrumental in guiding patient counseling, discerning the extent of depression in individuals, and even facilitating experts in formulating tailored recommendations. In a holistic sense, the potential exhibited by ChatGPT in harnessing extensive language models to enhance psychological interventions for depression, grounded in human feedback, is palpable. This potential extends to diverse medical domains, entailing intricate clinical reasoning, marking a pivotal stride in the evolution of advanced chatbot applications.

Although the efficacy of AIGC is theoretically promising, mental health professionals shoulder the responsibility of upholding human well-being as paramount—consequently, the current stage warrants abstention regarding chat-based assessments as professionally conclusive, pending substantiating evidence. Our study encapsulates its outcomes in guidelines to illuminate forthcoming researchers, developers, and practitioners, enhancing their grasp of mental health literacy pertinent to downstream tasks.

## Data availability statement

The original contributions presented in the study are included in the article/supplementary material, further inquiries can be directed to the corresponding author/s.

## Author contributions

YL: Writing – review & editing, Writing – original draft, Supervision, Project administration, Funding acquisition. XD: Writing – original draft, Visualization, Software, Methodology, Data curation. SP: Writing – review & editing, Investigation, Conceptualization. CZ: Writing – review & editing, Methodology.

## References

[1] Willner P Rose J Stenfert Kroese B Murphy GH Langdon PE Clifford C. Effect of the COVID-19 pandemic on the mental health of carers of people with intellectual disabilities. J Appl Res Intellect Disabil. (2020) 33:1523–33. doi: 10.1111/jar.12811 32885897

[2] Craske MG Meuret AE Ritz T Treanor M Dour H Rosenfield D. Positive affect treatment for depression and anxiety: A randomized clinical trial for a core feature of anhedonia. J Consulting Clin Psychol. (2019) 87:457–71. doi: 10.1037/ccp0000396 30998048

[3] Liu Y Shi J Zhao C Zhang C. Generalizing factors of COVID-19 vaccine attitudes in different regions: A summary generation and topic modeling approach. Digital Health 9:20552076231188852. 10.1177/20552076231188852 PMC10359653 37485330

[4] Chancellor S De Choudhury M. Methods in predictive techniques for mental health status on social media: a critical review. NPJ Digit. Med. (2020) 3:43. doi: 10.1038/s41746-020-0233-7 32219184 PMC7093465

[5] Chandra Guntuku S Buffone A Jaidka K Eichstaedt JC Ungar LH. Understanding and measuring psychological stress using social media. ICWSM. (2019) 13:214–25. doi: 10.1609/icwsm.v13i01.3223

[6] Eichstaedt JC Smith RJ Merchant RM Ungar LH Crutchley P Preoţiuc-Pietro D. Facebook language predicts depression in medical records. Proc Natl Acad Sci USA. (2018) 115:11203–8. doi: 10.1073/pnas.1802331115 PMC6217418 30322910

[7] Liu Y. Depression detection via a Chinese social media platform: a novel causal relation-aware deep learning approach. J Supercomput. (2024) 80:10327–56. doi: 10.1007/s11227-023-05830-y

[8] Devlin J Chang M-W Lee K Toutanova K. Bert: Pre-training of deep bidirectional transformers for language understanding. arXiv preprint (2018) arXiv:1810.04805.

[9] Wei J Tay Y Bommasani R Raffel C Zoph B Borgeaud S. Emergent abilities of large language models. arXiv preprint (2022) arXiv:2206.07682.

[10] Biswas SS. Role of chat GPT in public health. Ann BioMed Eng. (2023) 51:868–9. doi: 10.1007/s10439-023-03172-7 36920578

[11] Wu J Gan W Chen Z Wan S Lin H. Ai-generated content (aigc): A survey. arXiv preprint (2023) arXiv:2304.06632.

[12] Thirunavukarasu AJ Ting DSJ Elangovan K Gutierrez L Tan TF Ting DSW. Large language models in medicine. Nat Med. (2023) 29:1930–40. doi: 10.1038/s41591-023-02448-8 37460753

[13] Liu Y Ding X Chi M Wu J Ma L. (2024). Assessing the helpfulness of hotel reviews for information overload: a multi-view spatial feature approach. Info Technol Tourism. 26(1):59–87.

[14] McGowan A Gui Y Dobbs M Shuster S Cotter M Selloni A. ChatGPT and Bard exhibit spontaneous citation fabrication during psychiatry literature search. Psychiatry Res. (2023) 326:115334. doi: 10.1016/j.psychres.2023.115334 37499282 PMC10424704

[15] Bubeck S Chandrasekaran V Eldan R Gehrke J Horvitz E Kamar E. Sparks of artificial general intelligence: early experiments with GPT-4. arXiv preprint (2023) arXiv:2303.12712.

[16] Elyoseph Z Levkovich I. Beyond human expertise: the promise and limitations of ChatGPT in suicide risk assessment. Front Psychiatry. (2023) 14:1213141. doi: 10.3389/fpsyt.2023.1213141 37593450 PMC10427505

[17] Xu X Yao B Dong Y Gabriel S Yu H Hendler J. Mental-LLM: leveraging large language models for mental health prediction via online text data. Proceedings of the ACM on Interactive, Mobile, Wearable and Ubiquitous Technologies (2024) 8(1):1–32. doi: 10.1145/3643540 PMC11806945 39925940

[18] Gunning D Stefik M Choi J Miller T Stumpf S Yang G-Z. XAI—Explainable artificial intelligence. Sci Robot. (2019) 4:eaay7120. doi: 10.1126/scirobotics.aay7120 33137719

[19] Mangalathu S Hwang S-H Jeon J-S. Failure mode and effects analysis of RC members based on machine-learning-based SHapley Additive exPlanations (SHAP) approach. Eng Structures. (2020) 219:110927. doi: 10.1016/j.engstruct.2020.110927

[20] Liu Y. Depression detection via a Chinese social media platform: a novel causal relation-aware deep learning approach. J Supercomput. (2024) 80:10327–56. doi: 10.1007/s11227-023-05830-y

[21] Ríssola EA Losada DE Crestani F. A survey of computational methods for online mental state assessment on social media. ACM Trans Comput Healthcare. (2021) 2:1–31. doi: 10.1145/3437259

[22] Zhang T Yang K Ji S Ananiadou S. Emotion fusion for mental illness detection from social media: A survey. Inf Fusion. (2023) 92:231–46. doi: 10.1016/j.inffus.2022.11.031

[23] Ragheb W Moulahi B Aze J Bringay S Servajean M. Temporal Mood Variation: at the CLEF eRisk-2018 Tasks for Early Risk Detection on The Internet. In CLEF (Working Notes). (2018).

[24] Cho K van Merrienboer B Gulcehre C Bahdanau D Bougares F Schwenk H. Learning phrase representations using RNN encoder-decoder for statistical machine translation. arXiv preprint (2014) arXiv:1406.1078.

[25] Paul S Kalyani JS Basu T. Early detection of signs of anorexia and depression over social media using Effective machine learning frameworks. In CLEF (Working notes). (2018).

[26] Sadeque F Xu D Bethard S. UArizona at the CLEF eRisk 2017 Pilot Task: Linear and Recurrent Models for Early Depression Detection. In CEUR workshop proceedings (Vol. 1866) (2017) NIH Public Access. PMC5654552 29075167

[27] Wang Y-T Huang H-H Chen H-H. A neural network approach to early risk detection of depression and anorexia on social media text. In CLEF (Working Notes) (2018), 1–8.

[28] Wang X Chen S Li T Li W Zhou Y Zheng J. Depression risk prediction for Chinese microblogs via deep-learning methods: content analysis. JMIR Med Inform. (2020) 8:e17958. doi: 10.2196/17958 32723719 PMC7424493

[29] Poświata R Perełkiewicz M. (2022). OPI@LT-EDI-ACL2022: detecting signs of depression from social media text using roBERTa pre-trained language models, in: Proceedings of the Second Workshop on Language Technology for Equality, Diversity and Inclusion. Presented at the Proceedings of the Second Workshop on Language Technology for Equality, Diversity and Inclusion, Dublin, Ireland. pp. 276–82. Association for Computational Linguistics. doi: 10.18653/v1/2022.ltedi-1.40

[30] Liu N Du M Hu X. (2019). Representation interpretation with spatial encoding and multimodal analytics, in: proceedings of the twelfth ACM international conference on web search and data mining, in: Presented at the WSDM ‘19: The Twelfth ACM International Conference on Web Search and Data Mining, ACM, Melbourne VIC Australia. pp. 60–8. doi: 10.1145/3289600.3290960

[31] Lundberg SM Lee S-I. A unified approach to interpreting model predictions. Advances in neural information processing systems (2017) 30.

[32] Plumb G Molitor D Talwalkar AS. Model agnostic supervised local explanations. Advances in neural information processing systems. (2018) 31.

[33] Rajani NF McCann B Xiong C Socher R. Explain yourself! leveraging language models for commonsense reasoning. arXiv preprint (2019) arXiv:1906.02361. doi: 10.18653/v1/P19-1

[34] Liu Y Chi M Sun Q. Sarcasm detection in hotel reviews: a multimodal deep learning approach. *J Hospital Tourism.* (2024).

[35] Zogan H Razzak I Wang X Jameel S Xu G. Explainable depression detection with multi-aspect features using a hybrid deep learning model on social media. World Wide Web. (2022) 25:281–304. doi: 10.1007/s11280-021-00992-2 35106059 PMC8795347

[36] Belcastro L Cantini R Marozzo F Talia D Trunfio P. Detecting mental disorder on social media: a ChatGPT-augmented explainable approach. arXiv preprint. (2024). arXiv:2401.17477.

[37] Wang Y Inkpen D Buddhitha P. (2024). Explainable depression detection using large language models on social media data, in: Proceedings of the 9th Workshop on Computational Linguistics and Clinical Psychology (CLPsych 2024), .

[38] Bao E Pérez A Parapar J. Explainable Depression Symptom Detection in Social Media. arXiv e-prints. (2023) arXiv-2310. 10.1007/s13755-024-00303-9 PMC11379836 39247905

[39] Liu Y Ott M Goyal N Du J Joshi M Chen D. Roberta: A robustly optimized bert pretraining approach. arXiv preprint (2019) arXiv:1907.11692.

[40] Kim Y. Convolutional Neural Networks for Sentence Classification. (2014), arXiv:1408.5882.

[41] Zhou C Sun C Liu Z Lau FCM. A C-LSTM neural network for text classification. arXiv preprint (2015) arXiv:1511.08630.

[42] Liu Y Zeng Q Li B Ma L Ordieres-Meré J. Anticipating financial distress of high-tech startups in the European Union: A machine learning approach for imbalanced samples. J Forecasting. (2022) 41:1131–55. doi: 10.1002/for.2852

